# Obsessive–Compulsive Traits and Problematic Internet Use Are Increased Among Adults with Autism Spectrum Disorder: Is There a Role of Obsessive Doubts and Communication Impairment?

**DOI:** 10.3390/brainsci14121170

**Published:** 2024-11-22

**Authors:** Barbara Carpita, Benedetta Nardi, Francesca Parri, Gianluca Cerofolini, Chiara Bonelli, Cristina Gaia Bocchino, Gabriele Massimetti, Ivan Mirko Cremone, Stefano Pini, Liliana Dell’Osso

**Affiliations:** Department of Clinical and Experimental Medicine, Section of Psychiatry, University of Pisa, 67 Via Roma, 56126 Pisa, Italy; barbara.carpita@unipi.it (B.C.); francyparri@icloud.com (F.P.); gianlucacerofolini@gmail.com (G.C.); chiarabonelli.95@hotmail.it (C.B.); cristinbocc16@gmail.com (C.G.B.); gabriele.massimetti@unipi.it (G.M.); ivan.cremone@gmail.com (I.M.C.); stefano.pini@unipi.it (S.P.); liliana.dellosso@unipi.it (L.D.)

**Keywords:** autism spectrum disorder, obsessive–compulsive disorder, obsessive–compulsive spectrum, problematic Internet use disorder

## Abstract

Background: The link between autism spectrum disorder (ASD) and obsessive–compulsive disorder (OCD) and the complexity of their differential diagnosis has been vastly investigated. Growing attention has been paid to the presence of problematic Internet use (PIU) in autistic individuals. Studies assessing OCD traits in autistic individuals are scarce and even less take into account the role that this overlap may have on the development and maintenance of PIU. We aimed to investigate OCD features in ASD individuals and their association with autism severity and the prevalence of PIU, and the potential dimensions associated with a greater probability of PIU. Methods: a total of 46 participants with ASD and 53 controls were assessed with the Adult Autism Subthreshold Spectrum questionnaire and the Obsessive–Compulsive Spectrum—Short Version. Results: There were significantly higher OCD features in ASD participants along with important correlations between OCD and ASD dimensions and a higher prevalence of PIU in the ASD group. Participants with putative PIU reported greater scores on some ASD and OCD dimensions, the with Doubt and Non-verbal communication domains emerging as significant predictors of the presence of putative PIU. Conclusions: These results support the three-way link between ASD, OCD, and PIU, contributing to the hypothesis of a neurodevelopmental basis for those conditions.

## 1. Introduction

Autism spectrum disorder (ASD) is a neurodevelopmental disorder whose main symptomatic features are represented by persistent deficits in communication and social interaction and the presence of restricted and repetitive behaviors or interests. These symptoms usually develop from an early age and ultimately lead to a significant impairment in the socio-occupational functioning [1]. However, ASD manifestations may dramatically vary depending on the individual, and may or may not be associated with intellectual disability or language impairments [1]. While comorbidity with both mental and somatic disorder is very frequent among individuals with ASD, the diagnosis is not always simple, as often, especially in the milder forms, ASD presence may be hidden by the concomitant occurrence of other psychopathological conditions [2,3,4]. There is strong evidence that people with ASD are more likely to have anxiety disorders, mood disorders, psychotic disorders, and obsessive–compulsive disorders (OCDs), which can worsen symptoms of ASD, lead to ongoing discomfort, and increased behavioral issues [5,6,7,8,9,10,11,12,13,14]. In this population, anxiety and related deficits are likely to remain untreated and eventually deteriorate if a proper examination and diagnosis are not obtained [15]. OCD is also frequently reported in people on the autism spectrum, although repeated actions and intrusive, recurring thoughts are common in both conditions and make it difficult to distinguish between them [16,17,18,19,20,21,22].

OCD represents the sixth most common psychiatric disorder with an annual prevalence ranging up to 2.3% [23,24] and is characterized by the presence of compulsions and/or obsessions [1]. While obsessions and compulsions are the hallmarks of OCD and patients usually exhibit both types of symptoms, only one of these symptoms is necessary to be diagnosed with OCD. Obsessions are defined as persistent and frequent thoughts, urges, or impulses that cause discomfort or anxiety. Although the distinction can be blurry in some situations, these can be distinguished from delusions since they are usually seen as unrealistic or exaggerated and are often regarded as intrusive or ego-dystonic. Contrarily, compulsions are recurrent actions or thoughts that are often triggered by obsessions and are performed in an effort to avoid dreaded situations or lessen anxiety and distress [1]. Some examples of obsession–compulsion dyads are fears about germs or contamination and the urge to wash their hands unnecessarily, fear of intruders, repeatedly checking to make sure that doors and windows are locked, and believing that negative consequences will occur if things are not done perfectly, and redoing something until it feels “right”. The diagnosis of OCD usually occurs in adulthood, but numerous studies have shown how OCD develops starting from childhood and adolescence [25,26]. Although most clinical studies on OCD have concentrated on full-blown presentations, studies have shown that OCD can also manifest in a milder subsyndromal form, as with many other psychiatric disorders [27,28] that seem particularly frequent among specific groups of individuals, such as patients with ASD [23,27,29,30,31,32,33,34]. In this context, the link between ASD and OCD has been vastly investigated and documented. Notwithstanding some variability, the literature has broadly highlighted an overall double risk for ASD individuals to receive an OCD diagnosis during their lifetime when compared to the general population [10,33,35]. Despite this, making a diagnosis of OCD in ASD individuals is extremely complex due to many factors such as the presence of possible communication difficulties typical of ASD, difficulties in the recognition of their emotional and internal states, and a lack of insight regarding OCD symptoms [33,34,36,37]. One of the elements that poses the greatest difficulty in distinguishing the two conditions is the presence of restricted and repetitive interests of ASD individuals, as these can be confused with the obsessions and compulsions typical of OCD [33,38]. However, a major discriminant factor is represented by the ego-dystonic OCD repetitive behaviors, while in ASD, the ritualistic and repetitive behaviors appear to be ego-syntonic, reducing anxiety and usually bringing a sense of well-being [22,33,39,40].

Lastly, growing attention has recently been paid to the presence of problematic Internet use (PIU) in individuals who are on the autism spectrum. PIU is a catch-all term for a variety of repetitive, functionally impairing compulsive behaviors that have surfaced in the last ten or so years, including excessive online gaming, gambling, sexual behavior, shopping, video-streaming, and social media use. These behaviors were especially pertinent during the COVID-19 pandemic, when there are a high prevalence of digital technology use. Despite its growing relevance in the psychiatric field, PIU is not yet recognized as a disorder by the DSM-5-TR or ICD-11; however, the last edition of the DMS-5-TR recognizes Internet Gaming Disorder (IGD), which represents a form of PIU aimed at Internet gaming, as one of the emerging disorders that need further study. Such disorders are characterized by some typical elements of addictions such as loss of control, tolerance, withdrawal, and craving. In particular, the DSM-5-TR proposed that diagnostic criteria for IGD, aside from the excessive use of online games leading to significant impairment or distress, include the presence of at least five of the following: preoccupation with Internet games, withdrawal symptoms when not gaming, tolerance, unsuccessful attempts to reduce or control gaming, loss of interest in other activities, continued excessive use despite knowing it causes problems, deception about the extent of gaming, escaping or relieving negative emotions through gaming and significant consequences such as damaged relationships, job loss, and missed educational opportunities. Such symptomatology should persist for more than 12 months [1,41]. This condition seems to predominantly involve the new generations who have grown up in contact with technology [42].

Interestingly, it has been reported that OCD not only promotes the onset of PIU, but also shares several symptomatic domains and neurophysiological correlates. For example, failure in inhibitory control, cognitive inflexibility and an inefficient top-down regulation typical of OCD [43,44,45] has also been described in subjects with PIU [46,47]. Moreover, some PIU habits such as constantly checking emails or social media, or digital hoarding are quite akin to obsessive disorders like OCD, indicating a closer connection to anxiety or compulsive mechanism [48]. In this framework, numerous studies have documented a correlation between OCD and ASD, thanks to the presence of repetitive behaviors in both disorders [49,50,51]. Indeed, according to a major study conducted on the Danish population, subjects with OCD are four times more likely to acquire a second diagnosis of ASD, and those who were initially diagnosed with ASD are twice as likely to receive a second diagnosis of OCD [35]. Moreover, according to estimates, OCD prevalence varies between 4.9% and 37.2% in children and adolescents with ASD and between 7% and 24% in adults with ASD [21,52,53]. Conversely, individuals with OCD not only have a higher chance of meeting the diagnostic criteria for ASD [35,51], but they also exhibit noticeably more autistic characteristics, which exacerbates the disorder’s symptoms [51,54,55]. On the other hand, PIU seems to affect individuals with ASD more frequently and severely [56]. According to recent reports, people with ASD regularly overuse Internet and video games [57,58], spending roughly 62% more time on screen activities than they do engaging in any other non-screen activity. It was suggested that people with ASD were well suited to on screen activities because of their higher attention to detail, weak central coherence, and occasionally displayed visual skills [59]. On the other hand, through creative play, video game-playing subjects on the spectrum may circumvent their challenges and enhance their executive functioning [60]. In particular, the development of online connections, which are less constrained by emotional, social, and temporal limitations, may help patients with ASD overcome some of the communication and social interaction challenges that they face [61]. According to this viewpoint, some writers have even proposed that electronic media may sometimes be a useful tool for social connection in people with ASD, with benefits for cognitive growth and general well-being.

To date, studies assessing obsessive–compulsive traits in autistic individuals are relatively scarce and primarily focused on children and teenagers, and even fewer take into account the correlation that this overlap could have on the development and maintenance of a PIU. Nonetheless, these characteristics may be crucial in determining the course of psychopathology and the efficacy of therapy, even in adulthood. Within this framework, the aim of this study was to investigate the presence of obsessive–compulsive features in a sample of adult individuals with ASD and healthy controls (HCs), as well as their association with ASD severity. We also aimed to assess and compare the prevalence of PIU, as well as investigate potential obsessive or autistic dimensions statistically associated with a greater probability of a co-occurrent PIU.

## 2. Materials and Methods

### 2.1. Study Sample and Procedures

The sample was composed of 99 participants: 46 participants with a diagnosis of ASD and 53 healthy controls (HCs), recruited between September 2022 and March 2023.

The ASD group was enrolled from patients attending the University of Pisa Psychiatric Clinic, whereas the HC individuals were selected from medical and paramedical staff. Subjects under 18 and above 70 years old, with mental disabilities, persistent psychotic symptoms, and language or intellectual impairments that would jeopardize the exams were excluded. The absence of mental illnesses in HCs was verified using the Structured Clinical Interview for DSM-5, Research Version (SCID-5-RV) [62]. The diagnosis of ASD was made by trained psychiatrists specialized in neurodevelopmental disorders in adults, according to DSM-5-TR criteria, after a thorough clinical evaluation. Moreover, all ASD participants scored 70 or higher on the AdAS Spectrum questionnaire, which is the threshold for identifying subjects with full-blown ASD.

After receiving a thorough explanation of the procedures, the recruited individuals provided signed informed consent.

The total sample was made of 46 ASD subjects and 53 HC. The ASD group contained 23 (50%) males and 23 (50%) females and showed an overall mean age of 34.35 years (±11.51). The HC group contained 25 (47.2%) males and 28 (52.8%) females and showed an overall mean age of 38.47 years (±13.38). The two groups did not differ significantly for gender (X^2^ = 0.079; *p* = 0.779) nor for age (t = −1.649, *p* = 0.106) (see Table 1). Full IQ scores were not calculated.

Subjects belonging to the HC group obtained a mean score of 5.53 ± 4.53.

### 2.2. Measures

#### 2.2.1. Adult Autism Subthreshold Spectrum (AdAS Spectrum)

The AdAS Spectrum is a 160-item self-report instrument used to assess a wide variety of autistic symptoms in individuals without intellectual or language impairments. It is composed of seven domains investigating manifestations relating to Childhood and Adolescence, deficits in Verbal and Non-verbal communication, Empathy alterations, a tendency toward Inflexibility and adherence to routine, presence of Restricted interests and rumination and Hyper- and Hyporeactivity to sensory input. The AdAS Spectrum requires a score of at least 70 for identifying subjects with full-blown ASD, and 43 for determining the presence of significant autistic traits [63]. Excellent test–retest reliability, good internal consistency, and convergent validity with other dimensional measures of autism spectrum were reported in a validation study [64]. Moreover, as in previous research [65], in this study, we focused on one specific Item (n°66), which investigates the presence of PIU by asking the following question: “Do you spend a lot of time playing videogames or surfing on the Internet, to the extent of forgetting to do routine tasks?”. Specifically, individuals who supported AdAS Spectrum item number 66 were classified as potentially having PIU.

#### 2.2.2. Obsessive–Compulsive Spectrum—Short Version (OBS-SV)

The OBS-SV is a self-report questionnaire used for evaluating unique manifestations, temperamental features, and other clinical and subclinical components of OCD in addition to the prototypical symptoms of the disorder. The questionnaire is divided into six categories and two appendices, featuring a total of 139 items with binary answers. The domains involve the evaluation of manifestation related to the dimensions of Doubt, Hypercontrol, Temporal dimension, Perfectionism, Repetition and automation and Obsessive themes. The appendices include the assessment of the presence of obsessive traits during Childhood and adolescence and the tendency towards Impulsivity and loss of control. Excellent internal consistency, test–retest reliability, and considerable convergent validity with other OCD measures were reported in a validation study [27].

### 2.3. Statistical Analysis

We tested the normal distribution of data using the explore procedure, which showed that our variables were not normally distributed (age excluded), the median value did not fall within the confidence interval of means and the Kolmogorov–Smirnov and Shapiro tests were all significant. On this basis, we have chosen to use nonparametric tests for univariate comparisons and to apply the bootstrapping technique (number of samples = 1000) for multivariate comparisons (MANOVAs). Similarly, we have applied bootstrapping techniques to linear and logistic regressions as well.

When checking comparisons, the Bonferroni correction was used to reduce family-wise error rates.

Spearman’s correlation coefficient was employed to assess the pattern of correlations between the scores recorded on the two psychometric tools.

Afterwards, two models of linear regression analyses were performed using the AdAS Spectrum total as a dependent variable: the first providing as the independent variable the OBS-SV total score, and the second providing as independent variables all the OBS-SV domains scores. This was to identify the strongest predictors of a higher AdAS Spectrum total score.

Chi-squared tests were performed to compare by diagnostic group the proportion of putative PIU on the total sample and inside the ASD group discerning by gender.

MANOVAs were carried out to evaluate, in the ASD group, the effect of presence/absence of putative PIU on OBS-SV and AdAS Spectrum total and domain scores.

Lastly, in order to evaluate which OBS-SV and AdAS Spectrum domains were statistically predictive of PIU in the ASD sample, we performed two logistic regressions using the presence of putative PIU as dependent variable, while OBS-SV domains (first regression) and AdAS Spectrum domains (second regression), were used as independent variables.

All statistical analyses were performed with SPSS version 29.0.

## 3. Results

Table 2 reports Mann–Whitney results that showed how the ASD group scored significantly higher in all OBS-SV domains and in total compared to the HCs.

The correlation analysis showed that total OBS-SV and all its domain scores were significantly and positively correlated (medium to strong correlations) with all AdAS Spectrum domains and total score (see Table 3).

According to the linear regression analyses, the OBS-SV total score was shown to be a significant predictor of a higher AdAS Spectrum total score (b = 0.164, *p* < 0.001), as well as the OBS-SV Spectrum domains Doubt (b = 5.588, *p* = 002), Hypercontrol (b = 3.104, *p* = 0.005) and the Impulsivity appendix (b = 2.826, *p* = 0.009), whereas the Temporal dimension domain (b = −5.329, *p* = 0.022) emerged as a negative predictor of the AdAS Spectrum total score (see Table 4).

A further Chi-square analysis showed that the ASD group had a significantly higher frequency of putative PIU based on the positive responses at item 66 of the AdAS spectrum questionnaire compared to the HCs (see Table 5).

In the ASD diagnostic group, the comparison of the OBS-SV total score based on the presence/absence of PIUs is not significant (34.25 ± 17.64, mean rank = 27.13 vs. 26.65 ± 14.58, mean rank = 20.71; Mann–Whitney U = 332.500; *p* = 0.108).

The results from the first MANOVA analysis did not highlight a significant impact of the presence/absence of PIU on OBS-SV domains scores [Pillai’s Trace: V = 0.299, F (8,37) = 1.975, *p* = 0.077] even if the separate follow-up univariate ANOVAs on the outcome variables revealed a significant impact of presence/absence of PIU on the *Doubt* domain (5.50 ± 2.63 vs. 3.62 ± 2.12, F (1,44) = 7.265, *p* = 0.010) and *Childhood adolescence* appendix (6.10 ± 3.597 vs. 3.62 ± 3.32, F (1,44) = 5.883, *p* = 0.019). However, when applying the Bonferroni correction, even these differences would not be significant, with the required *p* value threshold for significant difference being <0.0055 (see Table 6).

The comparison on the total AdAS Spectrum score based on the presence/absence of PIU is not significant (89.20 ± 21.31, mean rank = 26.05 vs. 81.96 ± 17.15, mean rank = 21.54; Mann–Whitney U = 311.00; *p* = 0.258). The second MANOVA for comparison on AdAS domains scores show a significant difference [Pillai’s trace: V = 0.371, F(7,38) = 3206, *p* = 0.009]. The separate follow-up univariate ANOVAs on the AdAS domains revealed a significant impact of presence/absence of PIU on the Verbal communication (11.85 ± 3.39 vs. 9.42 ± 3.10, F(1,44) = 6.384, *p* = 0.015) and Non-verbal communication (17.60 ± 5.22 vs. 11.38 ± 4.74, F(1,44) = 17.809, *p* < 0.001) domains (see Table 7).

The results from a further logistic regression analysis, showed in Table 8a,b, highlighted the OBS-SV Doubt domain and AdAS Spectrum Non-verbal communication domain as significant predictors of the presence of putative PIU.

## 4. Discussion

Participants with autism scored significantly higher on the OBS-SV questionnaire, indicating a greater prevalence of obsessive symptoms in said population, in line with the available literature describing a greater prevalence of OCD and obsessive-like manifestation in ASD individuals [10,31,33,34]. Indeed, recent studies reported that ASD patients are two times more likely than the general population to receive an OCD diagnosis, and its overall predicted comorbidity incidence range between 6% and 37% [35,66,67,68]. To date, there are still few studies available that investigate the association between ASD and OCD as a comorbidity and the influence that each disorder has on the symptom severity of the other. Even fewer authors are taking into account adult patients, and those who do are often limited by the small sample size [69]. Further investigations are needed to better explain how one disorder can afflict another, worsening it and possibly evolving in new pathologic realities. Interestingly, alongside this higher prevalence, many authors highlighted an overlapping family history which suggests a common etiology for the two disorders. In particular, many studies have reported a bidirectional familiar link describing how the parents of ASD individuals reported higher levels of obsessive–compulsive symptoms and vice versa [70,71,72]. Our findings further confirm the link between autism and the obsessive–compulsive spectra showing that all OBS-SV domains and total scores were significantly correlated to all AdAS Spectrum domains and total score in line with the widely recognized symptomatologic overlap between the two disorders [31,34]. Moreover, according to our results, the OBS-SV total score and OBS-SV Doubt and Hypercontrol domains, as well as the Impulsivity appendix, appeared to be significant positive predictors of a greater AdAS Spectrum score. The first result confirms the evidence of a greater clinical severity in ASD individuals with comorbid obsessive symptoms [73]. Indeed, comorbid psychiatric disorders have been reported to exacerbate autistic symptomatology [5,7,8], ultimately aggravating impairments and complicating both diagnosis and treatment [21,73,74]. Particular interest should also be drawn to the OBS-SV *Doubt* domains, which investigates the tendency towards maladaptive rumination, known to play a central role in the development and maintenance of pathological obsessive symptoms [75,76,77]. Recent studies indicated that severe maladaptive rumination may be present in OCD patients, and that it has also been described to be connected with many obsessive features in non-clinical samples [78,79,80]. Maladaptive rumination appears to be one of the main symptoms of OCD, mediating the transition from naturally occurring thoughts to the exasperated tendency of OCD patients to overly focus on their ideas because they believe they are accountable for the consequences [75,81]. Similarly, a small but growing body of work indicates the presence of negatively valanced rumination in individuals with ASD, raising questions about how this should be conceptualized [77,82]. Furthermore, a growing body of research suggests that people with ASD struggle with inhibitory control, a deficit that fuels excessive rumination [82,83]. Therefore, individuals on the spectrum are expected to exhibit a greater tendency to ruminate about negative emotional events, given both the propensity for perseverance in ASD and a lack of inhibitory control [84]. The finding o*f Hypercontrol* domain scores as a positive predictor of greater autistic features is in line with a large body of research, which stressed that the main shared background for the link between OCD and ASD could be represented by cognitive inflexibility [85,86,87,88], characterized by the propensity to excessively focus on one’s own ideas, impairing the capacity to transition between different activities or actions [89,90,91].

Lastly, the result of the Impulsivity appendix being a positive predictor of greater ASD symptomatology is in line with the available literature that documents this domain in both disorders. Indeed, evidence of impulsive traits in OCD individuals have been widely reported [92,93,94,95,96], and many neurocognitive and neuroimaging studies suggested that OCD patient have disordered reward systems, which are linked to issues with impulse control and addiction [97,98,99,100]. Moreover, according to available data, individuals with OCD exhibit reduced resistance, control, and insight regarding their compulsive behaviors, especially those related to motor activities, characterizing the so called “impulsive compulsions,” which are compulsive behaviors carried out in an automatic or unplanned manner [101,102,103,104]. Similarly, deficiencies in motor inhibition have frequently been identified as a core feature of ASD, typified by impulsive, out-of-context, and premature motor behaviors [104,105,106,107,108] that ultimately result in the inability to ensure goal-directed, context-appropriate behaviors [108].

Some interesting and potentially controversial data that emerged from our results indicate the negative predictivity exerted by the *Temporal dimension* domain of the OBS-SV towards the total score of the AdAS Spectrum. This may be due to the tendency to focus and the global slowness in carrying out various activities typical of autistic individuals, experienced by them in an egosyntonic way, unlike the obsessive slowness of OCD individuals which, in an egodystonic way, causes discomfort and subjective suffering. This result is in line with recent studies that have introduced the “temporal theory” of ASD [109], which assumes that the surrounding world often changes too quickly to be addressed, perceived, and/or integrated in time by many individuals with ASD [110,111,112]. This primary temporal processing deficiency has many cascading consequences for the perceived symptomatology and behaviors [109,111,112]. Consequently, the slowing down of inputs, provided in the therapeutic context or actively implemented by well-functioning individuals, could have a positive influence on the perceived symptoms, acting as a mitigator [113,114,115,116,117].

Interestingly, ASD participants showed a greater frequency of putative PIU compared to HCs. These data align with the present literature, which highlighted that the use of Internet in ASD patients appears to be more represented, up to its pathological consequences [118,119,120]. Indeed, recent studies have shown how a greater presence of autistic traits is associated with a greater risk of PIU [119]. However, at the same time, many authors suggested that for individuals with ASD access to the Internet may represent an extremely useful mean of communication, facilitating the development of interpersonal exchanges and social relationships [121,122], ultimately paving the way for the possibility of a therapeutic application of Internet use [123,124].

Moreover, according to the results of logistic regression analyses, putative PIU was positively predicted by OBS-SV Doubt domain (and Childhood and Adolescence appendix only with a trend toward significance), as well as by AdAS Spectrum Non-verbal communication domain. A higher score on the Doubt dimension indicates the proneness towards ruminative thinking, including also the tendency to exhibit checking rituals in an attempt to control the maladaptive course of thought [75,81]. Its correlation with the presence of putative PIU is in line with recent studies that have suggested the presence of checking rituals as a risk factor for the development of PIU [125]. Analogously, the link between PIU and AdAS Spectrum Non-verbal communication domain may be related to the difficulties in interpersonal communication and social relations typical of autistic participants [1]. This feature becomes particularly relevant in high-functioning individuals who recognize their deficits in socio-emotional reciprocity and the management of social relationships and may implement adaptive strategies aimed at circumventing their shortcomings [126]. Noticeably, the link between Non-verbal communication and PIU was the only one confirmed by MANOVA when applying the Bonferroni correction, suggesting that this dimension should be considered the one most strongly related with PIU. In this context, the use of the Internet to undertake social relationships could appear particularly advantageous as the need to understand and reciprocate many of the verbal and non-verbal elements of communication would be eliminated [121,122,126]. Indeed, the presence of autistic traits may lead the individuals to prefer the use of the Internet for exchanges with other people, potentially resulting in a pathological use of it [59,61,118]. The use of the Internet may be used as a copying mechanism for the management of obsessive symptoms, within which the Doubting dimension constitutes a central nucleus [127,128] while simultaneously favoring the overcome of communication barriers intrinsic in individuals on the autistic spectrum, sometimes leading to the development of a PIU [59,119].

Our results should be seen in light of some limitations. Firstly, this study was designed as cross-sectional, and for this reason, we cannot draw conclusions regarding the temporal or causal connections present in the variables under analysis. Secondly, the psychometric instruments used were self-report questionnaires, and their score results may eventually be affected by an over- or under-estimation of symptoms by participants. Furthermore, this is a preliminary study that necessitates further investigations with more in-depth analyses to confirm our hypothesis. Lastly, the size of our sample is relatively small, restricting the generalizability of the results.

## 5. Conclusions

Our results highlighted a three-way link between ASD, OCD, and PIU, contributing to the hypothesis of a neurodevelopmental basis for those conditions.

## Figures and Tables

**Table 1:** Age and sex in the overall sample and comparison between diagnostic groups.

		ASD Mean ± SD	HC Mean ± SD	t	*p*
**Age**		34.35 ± 11.51	38.47 ± 13.38	−1.631	0.106
		**n (%)**	**n (%)**	**Chi-square**	***p***
**Sex**	**M**	23 (50%) ^a^	25 (47.2%) ^a^	0.079	0.779
	**F**	23 (50%) ^a^	28 (52.8%) ^a^		

Each superscript letter denotes a subset of categories whose column proportions do not differ significantly from each other at the 0.05 level.

**Table 2:** Comparison of OBS-SV scores among the diagnostic groups.

OBS-SV Scores	ASD Group Mean ± SD, Mean Rank	HC Group Mean ± SD, Mean Rank	U	*p* *
**Doubt**	4.43 ± 2.51, 72.21	0.98 ± 1.28, 31.59	243.50	<0.001
**Hypercontrol**	12.09 ± 6.31, 70.68	2.96 ± 3.87, 32.05	267.50	<0.001
**Temporal dimension**	4.48 ± 3.39, 60.33	1.28 ± 1.36, 41.04	744.00	<0.001
**Perfectionism**	1.78 ± 1.68, 65.71	0.72 ± 1.21, 36.37	496.50	<0.001
**Repetition and automation**	1.43 ± 1.67, 58.25	0.55 ± 0.99, 42.84	839.50	0.003
**Obsessive themes**	5.34 ± 5.08,65.27	1.07 ± 1.31, 36.75	516.50	<0.001
**Total Score**	29.96 ± 16.25, 71.86	7.57 ± 7.98, 31.03	213.50	<0.001
**Childhood and adolescence**	4.69 ± 3.63, 63.91	1.17 ± 1.42, 37.92	579.00	<0.001
**Impulsivity and loss of control**	3.83 ± 4.36, 64.84	0.62 ± 0.84, 37.12	536.50	<0.001

* When applying the Bonferroni correction, *p* is significant for *p* < 0.0056.

**Table 3:** Spearman’s correlations coefficients among AdAS Spectrum and OBS-SV domains and total scores in the total sample (ASD and HCs). All correlations were statistically significant (*p* < 0.05).

	Doubt	Hyperc.	Temp. Dim.	Perfect.	Repet. and Autom.	Obses. Themes	Tot. Score	Child./Adolesc.	Impuls.
**Child./** **Adolesc.**	0.589 **	0.588 **	0.289 *	0.387 **	0.235 *	0.393 **	0.605 **	0.393 **	0.470 **
**Verb. comm.**	0.625 **	0.560 **	0.282 *	0.360 **	0.294 *	0.474 **	0.619 **	0.368 **	0.490 **
**Non-verb. comm.**	0.670 **	0.581 **	0.295 *	0.477 **	0.314 *	0.513 **	0.660 **	0.434 **	0.554 **
**Empathy**	0.645 **	0.636 **	0.315 *	0.415 **	0.287 *	0.445 **	0.656 **	0.407 **	0.535 **
**Inflex. and routine**	0.600 **	0.675 **	0.371 **	0.522 **	0.304 *	0.407 **	0.659 **	0.405 **	0.350 **
**Restrict. interest and rum.**	0.619 **	0.734 **	0.460 **	0.569 **	0.352 **	0.492 **	0.744 **	0.505 **	0.390 **
**Hyper-/Hyporeact.**	0.568 **	0.555 **	0.216 *	0.409 **	0.204 *	0.312 *	0.557 **	0.323 *	0.398 **
**Total Score**	0.638 **	0.637 **	0.313 *	0.434 **	0.297 *	0.400 **	0.654 **	0.394 **	0.469 **

*: *p* < 0.05; **: *p* < 0.001.

**Table 4:** Bootstrap linear regression analysis with AdAS Spectrum total score as a dependent variable and OBS-SV total and OBS-SV domains as independent variables in the total sample.

	b	dist	SE	*p*	C.I. 95%	
					Lower Bound	Upper Bound
*constant*	13.037	0.088	4.431	0.008	4.658	22.013
**OBS-SV Total Score**	1.640	0.000	0.143	<0.001	1.364	1.924
*constant*	8.057	−0.536	3.967	0.065	0.461	16.104
**Doubt**	5.88	−0.063	1.706	0.002	1.743	8.641
**Hypercontrol**	3.104	0.095	1.113	0.005	1.197	5.486
**Temporal dimension**	−5.329	0.011	2.330	0.022	−10.298	−0.788
**Perfectionism**	0.503	−0.047	1.718	0.757	−3.187	3.447
**Repetition**	−4.773	−0.170	2.707	0.063	−10.022	0.0714
**OC Themes**	−0.630	0.048	1.207	0.584	−3.146	1.774
**Childhood**	1.119	0.013	1.494	0.401	−2.011	4.016
**Impulsivity**	2.826	−0.105	1.067	0.009	0.514	4.797

**Table 5:** Comparison of frequency of putative PIU (dichotomous item) between the ASD group and HC group.

		ASD n (%)	HC n (%)	Chi-Square	*p*
*PIU*	**Yes**	20 (43.5%)	3 (5.7%)	19.748	<0.001
	**No**	26 (56.5%)	50 (94.3%)		

**Table 6:** Separate ANOVA analyses following MANOVA with OBS-SV domains as dependent variables and presence/absence of PIU as independent variable.

Source	Dependent Variable	Type III Sum of Squares	df	Mean Square	F	*p*
**Corrected model**	Doubt	40.151	1	40.151	7.265	0.010 *
	Hypercontrol	15.556	1	15.556	0.385	0.538
	Temporal dimension	0.488	1	0.488	0.168	0.683
	Perfectionism	6.294	1	6.294	0.542	0.466
	Repetition	7.658	1	7.658	2.864	0.980
	OC Themes	86.208	1	86.208	3.523	0.067
	Childhood	69.785	1	69.785	5.883	0.019 *
	Impulsivity	1986.992	1	1986.992	3.169	0.082
**Intercept**	Doubt	939.281	1	939.281	169.968	<0.001 *
	Hypercontrol	6689.904	1	6689.904	165.545	<0.001 *
	Temporal dimension	145.879	1	145.879	50.406	<0.001 *
	Perfectionism	926.641	1	926.641	79.760	<0.001 *
	Repetition	100.180	1	100.180	37.468	<0.001 *
	OC Themes	1584.295	1	1584.295	64.745	<0.001 *
	Childhood	1067.003	1	1067.003	89.947	<0.001 *
	Impulsivity	24,697.990	1	24,697.990	39.396	<0.001 *
**PIU**	Doubt	40.151	1	40.151	7.265	0.010 *
	Hypercontrol	15.556	1	15.556	0.385	0.538
	Temporal dimension	0.488	1	0.488	0.168	0.683
	Perfectionism	6.294	1	6.294	0.542	0.466
	Repetition	7.658	1	7.658	2.864	0.098
	OC Themes	86.208	1	86.208	3.523	0.067
	Childhood	69.785	1	69.785	5.883	0.019 *
	Impulsivity	1986.992	1	1986.992	3.169	0.082
**Error**	Doubt	243.154	44	5.526		
	Hypercontrol	1778.096	44	40.411		
	Temporal dimension	127.338	44	2.894		
	Perfectionism	511.185	44	11.618		
	Repetition	117.646	44	2.674		
	OC Themes	1076.662	44	24.470		
	Childhood	521.954	44	11.863		
	Impulsivity	27,584.243	44	626.915		
**Total**	Doubt	1188.000	46			
	Hypercontrol	8514.000	46			
	Temporal dimension	274.000	46			
	Perfectionism	1440.000	46			
	Repetition	220.000	46			
	OC Themes	2678.000	46			
	Childhood	1606.000	46			
	Impulsivity	52,871.972	46			
**Corrected total**	Doubt	283.304	45			
	Hypercontrol	1793.652	45			
	Temporal dimension	127.826	45			
	Perfectionism	517.478	45			
	Repetition	125.304	45			
	OC Themes	1162.870	45			
	Childhood	591.739	45			
	Impulsivity	29,571.235	45			

* When applying the Bonferroni correction, *p* is significant for *p* < 0.0055.

**Table 7:** Bootstrap ANOVA analyses with AdAS Spectrum domains as dependent variables and presence of PIU as independent variable.

Source	Dependent Variable	Type III Sum of Squares	df	Mean Square	F	*p* *
Corrected Model	**Child./** **Adolesc.**	21.552	1	21.552	1.527	0.223
	**Verb. comm.**	66.582	1	66.582	6.384	0.015
	**Non-verb. comm.**	463.698	1	463.698	17.809	0.000
	**Empathy**	7.658	1	7.658	1.288	0.263
	**Inflex. and routine**	118.556	1	118.556	1.381	0.246
	**Restrict. interest and rum.**	5.662	1	5.662	0.242	0.625
	**Hyper-/Hyporeact.**	1.295	1	1.295	0.057	0.813
Intercept	**Child./** **Adolesc.**	7019.639	1	7019.639	497.233	0.000
	**Verb. comm.**	5115.713	1	5115.713	490.506	0.000
	**Non-verb. comm.**	9496.872	1	9496.872	387.285	0.000
	**Empathy**	2535.658	1	2535.658	426.412	0.000
	**Inflex. and routine**	20,744.991	1	20,744.991	241.722	0.000
	**Restrict. interest and rum.**	5932.096	1	5932.096	253.450	0.000
	**Hyper-/Hyporeact.**	2632.947	1	2632.947	115.197	0.000
PIU	**Child./** **Adolesc.**	21.552	1	21.552	1.527	0.223
	**Verb. comm.**	66.582	1	66.582	6.384	0.015
	**Non-verb. comm.**	436.698	1	436.698	17.809	**0.000**
	**Empathy**	7.658	1	7.658	1.288	0.263
	**Inflex. and routine**	118.556	1	118.556	1.381	0.246
	**Restrict. interest and rum.**	5.662	1	5.662	0.242	0.625
	**Hyper-/Hyporeact.**	1.295	1	1.295	0.057	0.813
Error	**Child./** **Adolesc.**	621.165	44	14.117		
	**Verb. comm.**	458.896	44	10.429		
	**Non-verb. comm.**	1078.954	44	24.522		
	**Empathy**	261.646	44	5.947		
	**Inflex. and routine**	3776.162	44	85.822		
	**Restrict. interest and rum.**	1029.838	44	23.405		
	**Hyper-/Hyporeact.**	1005.662	44	22.856		
Total	**Child./** **Adolesc.**	7681.000	46			
	**Verb. comm.**	5576.000	46			
	**Non-verb. comm.**	10,644.000	46			
	**Empathy**	2812.000	46			
	**Inflex. and routine**	25,417.000	46			
	**Restrict. interest and rum.**	7119.000	46			
	**Hyper-/Hyporeact.**	3670.000	46			
Corrected total	**Child./** **Adolesc.**	642.717	45			
	**Verb. comm.**	525.478	45			
	**Non-verb. comm.**	1515.652	45			
	**Empathy**	269.304	45			
	**Inflex. and routine**	3894.717	45			
	**Restrict. interest and rum.**	1035.500	45			
	**Hyper-/Hyporeact.**	1006.957	45			

* When applying the Bonferroni correction, *p* is significant for *p* < 0.0063.

**Table 8:** (**a**) Bootstrap logistic regression analysis with the presence of putative PIU as a dependent variable and OBS-SV domains as independent variables in the ASD sample. (**b**) Bootstrap logistic regression analysis with the presence of putative PIU as a dependent variable and AdAS Spectrum domains as independent variables in the ASD sample.

	b	dist	SE	*p*	C.I. 95%	
					Lower Bound	Upper Bound
(**a**)						
*constant*	−1.881	−10.798	90.624	0.039	−109.330	0.438
**Doubt**	0.337	4.373	39.755	0.060	−0.248	25.013
**Hypercontrol**	−0.188	−2.855	21.430	0.110	−24.805	0.156
**Temporal dimension**	−0.068	−3.487	72.087	0.795	−17.363	1.626
**Perfectionism**	0.013	−0.275	18.218	0.896	−1.344	8.518
**Repetition**	0.233	2.856	70.363	0.480	−2.585	11.481
**OC Themes**	0.020	0.363	21.654	0.854	−2.326	1.752
**Childhood**	0.365	5.540	42.185	0.022 *	−0.053	62.762
**Impulsivity**	0.044	0.082	14.450	0.622	−1.226	2.032
Cox and Snell R square = 0.290; Nagelkerke R square = 0.390.						
(**b**)						
*constant*	−6.570	−33.520	158.337	0.010 *	−605.508	1.666
**Child./** **Adolesc.**	−0.108	−2.953	20.379	0.574	−61.834	1.041
**Verb. comm.**	0.149	0.194	16.259	0.436	−10.225	22.332
**Non-verb. comm.**	0.501	5.296	26.718	0.010 *	0.269	89.750
**Empathy**	0.145	2.954	20.520	0.475	−1.692	30.315
**Inflex. and routine**	−0.170	2.645	14.905	0.050	−40.478	0.062
**Restrict. interest and rum.**	0.172	1.725	8.496	0.129	−0.150	35.539
**Hyper-/Hyporeact.**	−0.121	0.383	16.163	0.366	−28.160	4.918
Cox and Snell R square = 0.408; Nagelkerke R square = 0.548.						

* significant for *p* < 0.005.

## Data Availability

The raw data supporting the conclusions of this article will be made available by the authors, without undue reservation.
